# Dental Colleges

**Published:** 1882-12

**Authors:** 


### EDITORIAL, ETC.

---

*Dental Colleges.*—Dr. George Watt, the able editor of the *Ohio State Journal of Dental Science* in an editorial with the above title, very truly remarks that "formerly there were those who willingly made great sacrifices as teachers in the dental colleges, believing that, in the state of the profession at that time, it was their duty to do so without financial reward. But now in the advanced condition of the profession, such inducements do not exist, and similar sacrifices are not to be expected. The teachers must now receive financial compensation for their labors. The schools not endowed must hold out strong inducements to attract students. Since they supply the funds it is necessary to matriculate them without requiring them to understand even the common branches of an English education. Ichabod might be inscribed on the doors of any one of these colleges, not connected with Universities, that would not cheerfully receive all who apply, and graduate them without much regard to their attainments. Students would go elsewhere, and the colleges would die of inanition. As most dental students when matriculated, are wanting the mental training to make them apt scholars, and few of them have much knowledge of the studies to be pursued, the tuition in our dental colleges is too short. The average term of those, not departments of Universities, is about nineteen weeks. Deducting two weeks to get the pupils well at their studies at the commencement of the terms, two weeks for the holidays, and one week for the examinations at the end of each term, leaves fourteen weeks for study. It is not to be presumed that *the* students will devote much time to study during the vacations."

He then asks "are twenty-eight weeks, with all the advantages a college can afford, a sufficient time to require a knowledge of anatomy and oral surgery with dissections, physiology, histology, pathology, therapeutics, chemistry, and operative and mechanical dentistry? The result is that most of the students in the dental schools require a smattering of what is taught, and are usually graduated without much attention given to the evidences of their attainments, shown by their examinations. Is this the proper way to elevate and maintain the standing of a profession recognized as learned, and a branch of that engaged in the practice of the healing art?" He further states "there is little probability that dental schools not connected with Universities, would agree on and abide by any rule requiring a good English education of those received as pupils, and extend the time of pupilege to enable them to gain the requisite knowledge of the subjects taught. Were all dental colleges department of Universities, these difficulties might be more readily avoided. supported from the common fund they would be free from the strong inducements to receive students without preliminary examination, and grant diplomas to those not well qualified."

As Dr. Watt is well known to have been connected for many years with the faculty of a dental school, and everywhere acknowledged as one of the most enlightened members of the dental profession, his opinions on the subject of dental education are worthy of the highest consideration.

